# Concurrent detection of *cagA, vacA, sodB* and *hsp60* virulence genes and their relationship with clinical outcomes of disease in *Helicobacter pylori* isolated strains of southwest of Iran

**Published:** 2019-06

**Authors:** Mansour Amin, Ali Akbar Shayesteh, Amirarsalan Serajian

**Affiliations:** 1 Department of Microbiology, Faculty of Medicine, Ahvaz Jundishapur University of Medical Sciences, Ahvaz, Iran; 2 Research Center for Infectious Diseases of Digestive System, Ahvaz Jundishapur University of Medical Sciences, Ahvaz, Iran

**Keywords:** *Helicobacter pylori*, Multiplex polymerase chain reaction, Virulence genes, Chronic gastritis, Gastric disorders peptic ulcers

## Abstract

**Background and Objectives:**

*Helicobacter pylori* is a Gram-negative spiral-shaped bacterium that contaminates more than half of the world’s inhabitants, and infection with this bacterium is associated with some gastric disorders. Also, 5% to 10% of *H. pylori* genes are specific to this bacterium and many bacterial virulence factors fall into this group. The *cagA, vacA, sodB* and *hsp60* are among important virulence factors of *H. pylori*.

**Materials and Methods:**

A gastric biopsy specimen was taken from 341 gastric patients and cultivated on a Colombia agar plate, containing various antibiotics, such as vancomycin, amphotericin B, and trimethoprim & polymyxin B, and incubated for 3 to 10 days under microaerophilic conditions at 37°C. PCR was used to detect the *ureC, cagA, vacA, sodB* and *hsp60* genes.

**Results:**

In this study, 131 isolates were identified as *H. pylori*. The prevalence of *cagA, vacA, sodB* and *hsp60* were 74%, 100%, 92.4% and 96.2%, respectively. The correlation between the clinical forms of the disease and the virulence genes were analyzed by statistical tests and no significant correlation was found.

**Conclusion:**

The obtained results are similar to some studies conducted in different parts of the world and is different in other cases. This discrepancy is due to the difference in the type of gastric disorders, sample size and methodology.

## INTRODUCTION

*Helicobacter pylori* belongs to the class of Epsilonproteobacteria, order of Campylobacterales, and family of *Helicobacteriaceae* (1). It is a Gram-negative and spiral-shaped bacterium that contaminates more than half of the world’s inhabitants, and infection with this bacterium is associated with chronic gastritis, peptic ulcers, mucosa-associated lymphoid tissue (MALT), lymphoma, and gastric cancer (2–4). In 1994, the World Health Organization (WHO) categorized *H. pylori* as carcinogen type 1 due to its role in gastric cancer (5, 6). Factors that cause *H. pylori*-associated diseases include host susceptibility, environmental factors, and bacterial virulence factors (6–9). Out of the 1600 *H. pylori* genes, 5% to 10% are specific to this bacterium and many bacterial virulence factors fall into this group (10). Virulence factors are used as an important tool in determining the clinical outcomes of the disease (11, 12).

The *cagA* is an important virulence factor in *H. pylori*, located at the end of the cag pathogenicity island, and is responsible for encoding the CagA protein. The molecular weight of the CagA ranges from 120 to 140 kDa (13, 14). The cag pathogenicity island has about 30 genes and is responsible for encoding the components of type IV secretion systems. The function of this system is to inject the Cag protein into the host cells (7, 15). In Western countries, individuals with the Cag positive strains of *H. pylori* have a higher risk of developing gastric ulcer or gastric cancer than the Cag negative strains (16, 17). The reported prevalence of *cagA* in Iran was 65.9%, while this prevalence was 60%–70% in Western countries, and more than 95% in Eastern countries (7, 18).

The CagA protein is highly immunogenic and is activated by phosphorylation in the host cell cytoplasm and causes morphological and functional changes in the host cell (10, 19). This protein promotes cell proliferation and differentiation and reduces apoptosis, which is a condition beneficial for induction of tumor cells. In addition, CagA induces cell polarity, morphological changes, and epithelial-mesenchymal cellular changes, which stimulate gastric cancer (15). Several studies have shown that the presence of *cagA* in *H. pylori* isolates increases the chances of developing gastrointestinal diseases (1, 5, 7, 18, 20).

The second most effective toxin in the pathogenicity of *H. pylori* is VacA toxin, encoded by the *vacA* (18). Almost all *H. pylori* strains harbor this gene, but it is expressed in only 50% to 60% of the strains (21, 22). Several studies have shown that the most important role of VacA in the pathogenesis of the disease is exerted by inducing the autophagy, which results in the development of the vacuole and the possibility of intracellular survival of bacteria (23, 24).

Following the entry of VacA into the cell, toxin is transmitted to the cell mitochondria and induces apoptosis. Subsequent to apoptosis, large intracellular vacuoles will be formed in the cytoplasm of the epithelial cells of the stomach (2, 25). Toxin also induces a proinflammatory effect on T-cells that is mediated by the activation of NF-κB, resulting in an increase in the secretion of IL-8 (26).

Another important factor in maintaining and surviving *H. pylori* is the superoxide dismutase (SOD) enzyme encoded by the *sodB* (27). This enzyme is important for the detoxification of reactive oxygen species (ROS), produced by neutrophils and monocytes during the inflammatory process, and it protects the bacteria. The SOD enzyme catalyzes the radical superoxide into hydrogen peroxide and molecular oxygen (13).

The Hsp60 (Heat Shock Protein 60) protein is one of the most abundant proteins in *H. pylori* and acts as a molecular chaperone (28, 29). Molecular chaperone, such as Hsp60, protect unfolded proteins against accumulation of acid (30). The Hsp60 activates interleukin 6 production by macrophages and plays an important role in the host defense mechanism. Limited studies have been conducted on Hsp60 and superoxide dismutase, but they all emphasized the role of these 2 proteins in the pathogenesis of bacteria (27, 30, 31). The aim of this study was to design a multiplex PCR for simultaneous detection of *H. pylori* virulence genes and to confirm the relationship of these genes with the clinical outcomes of *H. pylori* infections.

## MATERIALS AND METHODS

### Sample collection, culturing, and isolation of strains.

From the beginning of July 2015 until the end of June 2016, all stomach biopsies obtained from the patients who referred to the Gastroenterology Departments of Imam and Mehr Hospitals of Ahvaz were collected, stored on ice, and transferred to the Department of Microbiology, Faculty of Medicine, for bacteriological tests. The samples were grown in sterile tubes, containing BHI broth (Merck-Germany) and inactivated fetal bovine serum (Baharafshan-Iran). Then, samples were homogenized on a sterile slide using a scalpel blade and were cultured on a Colombia agar (Merck-Germany), containing 10% of freshly defibrinated sheep blood, supplemented with vancomycin (10 mg/L), amphotericin B (4 mg/L), trimethoprim (5 mg/L), and polymyxin B (10 mg/L) antibiotics (Sigma, USA) for 3 to 10 days under the microaerophilic conditions and incubated at 37°C (32). Culture media were examined after 72 hours for colony formation. Also, to confirm the *H. pylori*, the colonies were stained and checked for catalase, oxidase, urease (33). Then, from the confirmed strains, suspensions of BHI broth were prepared with 30% sterilized glycerol and 10% sterilized, heat inactivated fetal calf serum, and stored at −80°C (32). Before the endoscopy, to record the data, the patients were asked to complete a questionnaire.

### Ethical approval.

This study was approved by the ethics committee of Ahvaz Jundishapur University of Medical Sciences. (No. IR. AJUMS.REC.1395.581).

### DNA extraction.

DNA was extracted from the isolated colonies using a kit as instructed by the manufacturer (Roche-Germany). The extracted DNA was stored at −20°C until further use (6, 34).

### The amplification of *(ureC) glmM* gene.

The identity of the isolates was confirmed using the amplification of *(ureC) glmM* gene by PCR. The master mix consists of 800 μM dNTPs, 2.5 mM MgCl_2_, 50 mM buffer, 2.5 unit of Taq enzyme, and 1 μL of template in a total volume of 20 μL. The PCR cycling was as follows: an initial denaturation step at 94°C for 5 minutes, 40 cycles of denaturation at 94°C for 60 seconds, annealing at 55°C for 90 seconds, and extension at 72°C for 120 seconds. The final extension was performed at 72°C for 7 minutes. After the end of the reaction, PCR products (294bp) were electrophoresed on 1.5% agarose gel, containing ethidium bromide (8).

### Multiplex PCR method for concurrent detection of *sodB, hsp60, vacA* and *cagA* virulence genes.

To determine the simultaneous prevalence of the genes examined, a multiplex PCR was designed according to the annealing temperature of the genes. Multiplex PCR steps were as follow: (1) an initial denaturation for 5 minutes at 95°C; (2) 34 cycles, including 1 minute at 94°C, 1 minute primer annealing at 55°C, and extension for 1 minute at 72°C; (3) the final extension took place at 72°C for 10 minutes. Then, PCR products were electrophoresed on 1.5% agarose gel for 40 minutes at 130 volts with ethidium bromide. The master mix of this method includes 2.5 μL of PCR buffer, 1 μLl of dNTPs, 1 μL of MgCl_2_, 0.4 μL of Taq polymerase, 4 μL of template, and 0.2 μL of each of the primers. The final volume of the reaction was 25 μL (19, 27). Standard strain (ATCC26695) was used as a positive control. The name of the primers, the size of products, and the name of the genes examined are listed in Table 1.

**Table 1.** Product size and primers used for amplification of the genes

**Gene name**	**Primer sequence**	**Product size (bp)**	**Reference**
*glmM*	F-5′AAGCTTTTAGGGGTGTTAGGGGTTT3′ R-5′AAGCTTACTTTCTAACACTAACGC3′	294	5
*cagA*	F-5′GATAACAGGCAAGCTTTTGAGG3′ R-5′CTGCAAAAGATTGTTTGGCAG3′	349	35
*vacA*	F-5′CAATCGTGTGGGTTCTGGAGC3′ R-5′GCCGATATGCAAATGAGCCGC3′	678	27
*hsp60*	F-5′GCTCCAAGCATCACCAAAGACG3′ R-5′GCGGTTTGCCCTCTTTCATGG3′	603	27
*sodB*	F-5′GCCCTGTGGCGTTTGATTTCC3′ R-5′CATGCTCCCACACATCCACC3′	425	27

### Statistical analysis.

The data were analyzed using SPSS19. Pearson chi-square and Fisher’s exact tests were used to assess the relationships between the virulence genes and clinical forms of the disease. Also, the logistic regression test was used to determine the association of each form of the disease with virulence genes alone. P value of <0.05 was considered statistically significant.

## RESULTS

In this descriptive and analytical cross sectional study, 341 gastric antral biopsy samples from GI patients who referred to the GI Departments of Imam and Mehr hospitals in Ahvaz were evaluated. Of 341 endoscopic specimens, 131 isolates of *H. pylori* (38.4%) were isolated by culture, of which 70 (53%) were from males and 61 (47%) from females. The age range of the male patients was 16–84 years and that of females was 24–90 years. The grown colonies of the *H. pylori* were very fine, gray, transparent, and relatively convex. The isolated strains were identified using biochemical tests. Spiral Gram-negative isolates, positive for urease, oxidase and catalase tests, were preliminary identified as *H. pylori*.

### PCR results for detection of *ureC (glmM)* gene.

**Fig. 1.**
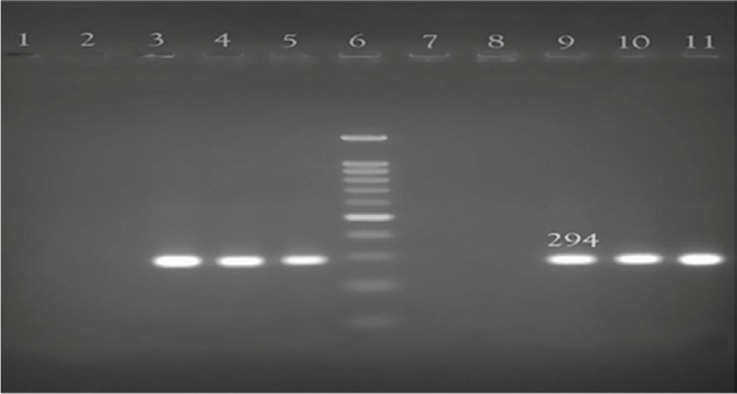
Agarose gel electrophoresis of PCR products of *glmM* gene. lane 6, 1000 bp DNA ladder, lanes 3, 4, 9, 10, 11 *H. pylori* isolates. lane 1 negative control. lane 5 positive control, lanes 2, 7, 8 non *H. pylori bacteria*

At first, the DNA of all isolates was extracted, and PCR was performed to detect the *glmM* gene. After PCR amplification, all isolates created a 294 bp fragment, thus confirming that all 131 isolates were *H. pylori* (Fig. 1).

For purification of bacteria from primary colonies, subculture was performed on antibiotic-free Columbia agar, containing fresh sheep blood. The clinical diagnosis of the patients, by endoscopy and pathology, was gastritis, peptic ulcer, duodenal ulcer, and gastric cancer. The relationship between age and sex of patients with clinical forms of *H. pylori* disease was studied using Chi-square and Fisher’s exact tests. Only the patient’s age and clinical forms of the disease were correlated (P value = 0.015). However, no significant relationship was found between the clinical forms of the disease and the sex of the patients (P value = 0.098). Moreover, the highest prevalence of gastric cancer was seen at age older than 70 years, and the highest incidence of gastritis was found in those aged 24–68 years.

According to the results of the study, the prevalence of gastritis was higher in females than in males, and the prevalence of gastric ulcer and duodenal ulcer was much higher in males than in females.

### Multiplex PCR results for detection of *sodB, hsp60, vacA* and *cagA* virulence genes.

**Fig. 2.**
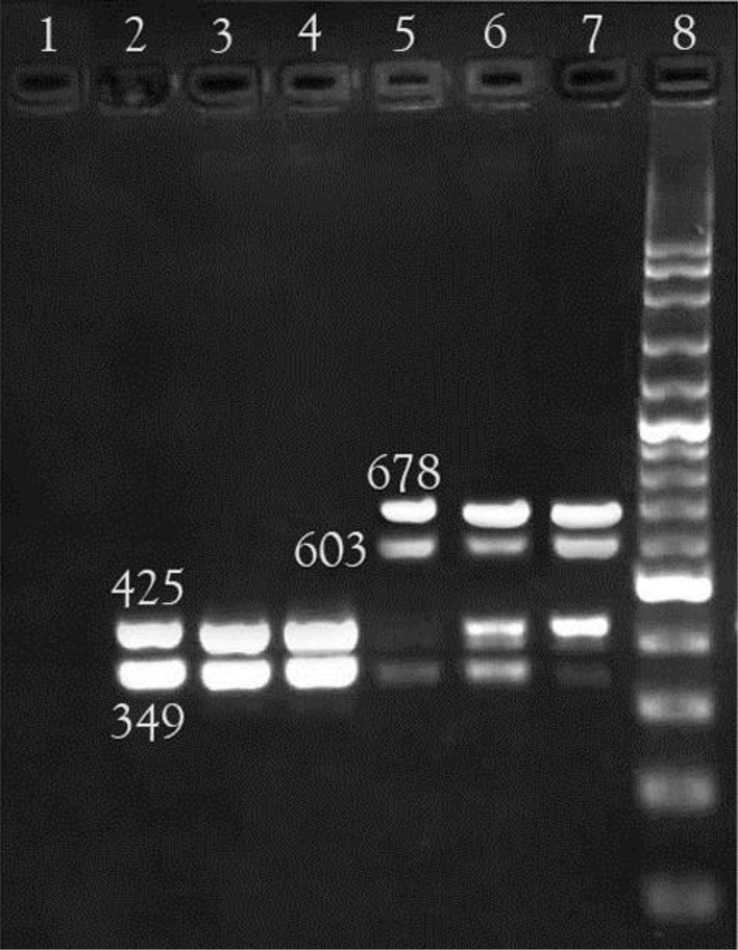
Agarose gel electrophoresis of multiplex PCR products. lane 8 1000 bp DNA ladder, lane 1 negative control, lane 6 positive control. 349, 425, 603 & 678 bp bands indicate *cagA, sodB, hsp60* & *vacA* genes.

To determine the virulence genes, *cagA, vacA, hsp60* and *sodB*, multiplex PCR experiments were designed, according to their specific annealing temperature, and the reaction was performed using the primers specified in Table 1, and the amplification of these genes created 349 bp, 678 bp, 603 bp, and 425 bp fragments, respectively. Then, the fragments were identified using 1.5% agarose gel electrophoresis. After performing multiplex PCRs, the prevalence of *cagA, vacA, hsp60* and *sodB* was found to be 74%, 100%, 96.2% and 92.4%, respectively (Fig. 2).

In this study, the relationship between clinical forms of the disease and *cagA, vacA, hsp60* and *sodB* was studied and approved using Chi-square and Fisher’s exact tests. Based on the results of these tests, no significant relationship was found between the clinical forms of the disease and *cagA, hsp60* and *sodB* (P value = 0.211, 0.214 and 0.452, respectively).

Due to the presence of *vacA* in all the examined isolates, the prevalence of this gene was similar in different clinical forms. Based on the results of statistical analysis, there was no significant correlation between virulence genes and clinical forms of the disease. However, the highest prevalence of *cagA* was found in the isolates from patients with ulcer and gastric cancer and the highest prevalence of *sodB* was seen in the isolates from patients with gastric cancer. Also, the lowest prevalence was found in the isolates of duodenal ulcer. Moreover, the highest prevalence of *hsp60* was found in the isolates from gastric cancer and gastric ulcer and the lowest prevalence in the isolates from duodenal ulcer.

### Determining the relationship between each form of the disease and virulence genes.

In this study, logistic regression test was used to examine the relationship between *H. pylori* virulence genes with each form of the disease. The results of the logistic regression test showed no significant relationship between gastritis, gastric ulcer, duodenal ulcer, and gastric cancer with the studied genes. Also, no significant difference was found in the chance of having these diseases in infected patients with isolates positive for these genes as well as isolates with no genes.

## DISCUSSION

Bacterial virulence factors are used as an important tool in determining the clinical outcomes of the disease; therefore, clinical isolates of *H. pylori* can be classified based on these factors (11, 12). Among the bacterial virulence factors involved in the pathogenesis of the bacterium are the *cagA, vacA, sodB* and *hsp60* genes. Multiplex PCR uses multiple primer sets within a single PCR mixture to produce amplicon of varying sizes and specific sequences of DNA. This PCR variant targets several genes in a single test. Using a sample design of the method in this study, the prevalence of *cagA, vacA, sodB* and *hsp60* was determined as 92.4%, 96.2%, 100% and 74%, respectively. In Western countries, 60%–70% of isolates are the *cagA* positive strains of *H. pylori*, while reports from East Asian countries show that more than 90% of isolates, regardless of the clinical form of the disease, are *cagA* positive (7, 18). According to the Saribasak and Ryberg studies, 78% and 82% of isolates from Turkey and Sweden have the *cagA* gene, but in the isolates studied by Edith Vega and Latifinavid, the prevalence was 40.8% and 65.9%, respectively (14, 18, 25, 27). The prevalence of the *cagA* gene in the present study was 74%, which is similar to a previous study in Iran (65.9%), but is different compared to some other studies in the rest of the world. The difference in the frequency of the *cagA* gene in different parts of the world can be due to the high ability of this bacterium to alter and diversify genetically in different geographical areas. The reason for the large variety of *H. pylori* is related to the genetic recombination, which is repeated frequently in the bacterial structure. In addition, the presence of mixed infections provides the possibility of genetic exchange between different strains.

The results of this study showed that all isolated strains of *H. pylori* harbor this gene, regardless of the clinical form of the disease. Therefore, the statistical analysis was not clear to determine the association of this gene with the clinical forms of the disease. The prevalence of the *vacA* gene in isolates of *H. pylori* in the Ferreira and Edith Vega studies was reported as 99.4%, 100% respectively, which is consistent with the results of this study, while the prevalence in the Owen and Farshad studies was 78% and 56.92%, respectively (8, 9, 25, 35).

The *hsp60* gene was found in 96.2% of isolates of this study. Mendoza and Ryberg have reported the prevalence of this gene to be 100% and 82%, respectively, indicating a high prevalence of this gene in isolates examined in different parts of the world (27, 30).

The last gene examined in this study was the *sodB* gene, which showed a prevalence of 92.4% in the isolates examined. There is not much study on this gene in the literature. One of the few studies on this gene was the study of Ryberg in Sweden that reported a prevalence of 72% for *H. pylori* strains, which is not consistent with the prevalence found in the present study (27).

Furthermore, the statistical analysis done in this study has not shown any association between clinical forms of gastritis, gastric ulcer, duodenal ulcer, and gastric cancer with these genes. However, the results of the present study showed that some of these genes are more common in some types of gastric diseases than other clinical forms. According to studies performed by Rasheed in Pakistan and Farshad in Iran, there is no significant correlation between the *cagA* virulence factor and various clinical forms of the disease, while Shiota found an association between the *cagA* and atrophy and gastric cancer (4, 35, 36). In addition, Saribasak believes that there is a link between the *cagA* and the clinical forms of gastric ulcer and gastric cancer (14). Vaziri also confirmed the relationship between the gene and the clinical form of gastritis (6).

In the present study, the prevalence of *hsp60* in isolates from gastric cancer and gastric ulcer was more than gastritis and duodenal ulcer, but this relationship was not confirmed by statistical methods. In the present study, the *sodB* positive isolates are more prevalent in patients with gastric cancer compared to other forms of the disease, but there is no significant relationship between this gene and various forms of the disease by statistical methods. The prevalence of the genes examined in this study was different compared to the prevalence reported in some studies in Iran and in other parts of the world, but it is similar to others. The reason for this discrepancy is the geographic differences of the strains studied, the sample size, the differences in clinical forms of the disease, and the primers designed for the PCR.

## CONCLUSION

The results of this study showed that different clinical forms of the disease did not have a significant relationship with virulence genes in *H. pylori* isolates, and these genes could not be used as a marker to determine the outcomes of infection with *H. pylori* in the study area. However, the prevalence of some of these genes in certain forms of *H. pylori* infection was more than other forms. Thus, it can be concluded that there is no relationship between the sex of the patients and the clinical forms of infection. On the other hand, a significant correlation was found between the age of the patients and various clinical forms of the disease. The results of the present study have indicated a higher prevalence of gastric cancer in older ages and a higher prevalence of gastritis in younger ages. It seems that despite the fact that the genes in the present study are not related to the clinical forms of *H. pylori* infections, this issue will continue to be the subject of controversial debate and discussion in scientific communities. However, in all *H. pylori* infections, in addition to the virulence factors analyzed in this study, other factors, such as environmental, host genetic, and other bacterial virulence have contributed to the clinical manifestation of the disease. Therefore, large-scale studies should be conducted in different geographic regions of Iran to clarify this issue.
